# Revisiting the Classification of Percid Perhabdoviruses Using New Full-Length Genomes

**DOI:** 10.3390/v12060649

**Published:** 2020-06-16

**Authors:** Laurane Pallandre, Dongsheng Luo, Claudette Feuvrier, François Lieffrig, Françoise Pozet, Laurent Dacheux, Laurent Bigarré

**Affiliations:** 1 Laboratory of Ploufragan-Plouzané-Niort, French Agency for Food, Environmental and Occupational Health & Safety, Technopole Brest Iroise, 29280 Plouzané, France; laurane.pallandre@anses.fr; 2 Institut Pasteur, Unit Lyssavirus Epidemiology and Neuropathology, 28 rue docteur Roux, 757240 Paris, France; dongsheng.luo@pasteur.fr (D.L.); laurent.dacheux@pasteur.fr (L.D.); 3 CAS Key Laboratory of Special Pathogens and Biosafety, Wuhan Institute of Virology, Chinese Academy of Sciences, Wuhan 430071, China; 4 University of Chinese Academy of Sciences, Beijing 100049, China; 5 Departemental Laboratory of Analyses of Jura, 39802 Poligny, France; cfeuvrier@jura.fr (C.F.); fpozet@jura.fr (F.P.); 6 CER Groupe, Laboratory of Fish Pathologies, 6900 Marche-en-Famenne, Belgium; F.Lieffrig@cergroupe.be

**Keywords:** *Rhabdoviridae*, *Perhabdovirus*, fish, percid, phylogenetic analysis, virus detection, complete genome, nucleoprotein

## Abstract

Perhabdoviruses are a threat to some freshwater fish species raised in aquaculture farms in Europe. Although the genetic diversity of these viruses is suspected to be high, the classification of isolates is still in its infancy, with just one full-length genome available and only partial sequences for a limited number of others. Here, we characterized a series of viruses isolated from percids in France from 1999 to 2009 by sequencing the nucleoprotein (N) gene. Four main clusters were distinguished, all related at varying levels of similarity to one of the two already-recognized species, namely *Perch perhabdovirus* and *Sea trout perhabdovirus*. Furthermore, we obtained the complete genome of five isolates, including one belonging to *Sea trout rhabdovirus*. The analysis of the complete L genes and the concatenated open reading frames confirmed the existence of four main genetic clusters, sharing 69 to 74% similarity. We propose the assignation of all these viral isolates into four species, including two new ones: *Perch perhabdovirus 1*, *Perch perhabdovirus 2*, *Sea trout perhabdovirus 1* and *Sea trout perhabdovirus 2*. In addition, we developed new primers to readily amplify specific portions of the N gene of any isolate of each species by conventional PCR. The presence of such genetically diverse viruses in France is likely due to divergent viral populations maintained in the wild and then introduced to experimental facilities or farms, as well as via trade between farms across the European continent. It is now urgent to improve the identification tools for this large group of viruses to prevent their unchecked dissemination.

## 1. Introduction

Over the last few decades, the aquaculture of percids (perch, *Perca fluviatilis* and pike-perch, *Sander lucioperca*), has intensified in Europe [1]. Consequently, the transfer of living biological material between regions and countries has increased both for research purposes and for trade. However, little attention has been given to the concomitant dissemination of fish pathogens, including viruses of the *Perhabdovirus* genus (*Rhabdoviridae* family). Since the 1980s, various perhabdoviruses have caused losses in percids and other freshwater species in farms or experimental facilities in European countries [2,3,4,5,6,7,8,9,10,11,12]. Infected fish often exhibit abnormal swimming behavior and lethargy, as well as hemorrhaging at the base of the fins. The disease has been reported in fry, juveniles and adults, with high mortality rates in some cases. Histopathology of affected juvenile perch reveals necrotic cells in the liver, spleen hematopoietic tissue and intestinal lamina propria, as well as congestion in the central nervous tissue. 

Perhabdoviruses can be isolated using cell cultures inoculated with infected internal organs, e.g., the anterior kidney, liver, spleen and brain from juveniles [10]. Their genome is made up of single-stranded negative RNA (ssRNA) of about 11.5 kb encoding the five canonical genes of rhabdoviruses in the following order: nucleoprotein (N), polymerase-associated phosphoprotein (P), matrix (M), glycoprotein (G) and RNA-dependent RNA polymerase (L) [13]. Among the three viral species recognized by the International Committee on Taxonomy of Viruses (ICTV; talk.ictvonline.org), members of two have been found to infect percids: *Perch perhabdovirus* (e.g., perch rhabdovirus, PRV) and *Sea trout perhabdovirus* (e.g., lake trout rhabdovirus, LTRV). Members of a third species (*Eel virus European X*) have never been isolated in percids, but the virus (EVEX) has been fully sequenced [13,14]. To date, only one PRV isolate has been completely sequenced, and about half of the genomes of two members of *Sea trout perhabdovirus*, namely LTRV and Swedish sea trout virus (SSTV), are available [13,15,16]. Other viral isolates have been partially sequenced, focusing on the partial N or L genes or the complete G open reading frame (ORF) [2,3,6,12]. Using these data, four (A-D) and two (E-F) genogroups have been distinguished within the *Perch perhabdovirus* and *Sea trout perhabdovirus* species, respectively. However, it is unclear if these genogroups are genuinely distinct or represent different species. To date, species demarcation is based on sequences of the L gene, which are not available for most viral isolates. Clearly, more genomic data, for instance complete genes or complete genomes, are needed to define the classification criteria for percid perhabdoviruses.

From a collection of percid perhabdoviruses mostly isolated in France, we obtained new sequences of the N ORF and a series of complete genomes from representatives of the genetic diversity known to date, which made it possible to carry out a more precise phylogenetic analysis of this virus genus.

## 2. Materials and Methods

### 2.1. Virus Isolation

All viruses in this study were isolated from perch or pike-perch. Except for one isolate (16/65) from Belgium, all the newly analyzed isolates originated from fish sampled in France (Table S1). Unfortunately, epidemiological information was not available for many isolates. One of the isolates (PRV) was the same as the one used by other authors to obtain a complete genome sequence [13].

Pooled organs (brain, kidney, spleen) from 5 to 10 fish or 1 g of pooled larvae were used to inoculate cell cultures. The organs were ground in a mortar with Fontainebleau sand and diluted to 1:10 with MEM (Glasgow medium, Sigma-Aldrich, St-Louis, USA). After centrifugation for 15 min at 2500 g at 4 °C, the supernatant of the homogenized organs was diluted to 1:10 and 1:100, and 0.1 mL of each dilution was inoculated onto duplicated monolayers of the cell cultures maintained with MEM and supplemented with 10% fetal bovine serum. For most viruses, the cultures were conducted on BF2 (bluegill fibroblast) and RTG2 (rainbow trout gonad) cell lines incubated at 14 °C for 7 days. Only virus 16/65 was cultured on an EPC (epithelioma paposum cyprini) cell line. Viral cytopathic effects (CPE) were recorded daily over the following week. A second passage in each cell line was done using aliquots of each dilution as inocula. When CPE were observed, the supernatant was collected and stored at −80 °C. CPE consisted of scattered foci of rounded and refringent cells; the foci then grew larger, with cell lysis spreading centrifugally, leading to the total destruction of the cell layers after 3 to 4 days.

### 2.2. Nucleic Acid Extraction for RT-PCR Detection

At the time of this study, no naturally infected tissue was available. Only virus-infected cell culture supernatants and farmed pike-perch larvae were available. Pike-perch larvae tested negative by cell culture and by the new specific PCRs (see below) were used to provide total tissues allegedly free of perhabdoviruses. For both types of material, total nucleic acids (NA) were extracted with a Nucleospin Virus kit (Macherey-Nagel, Duren, Germany) with minor modifications as previously described [17]. However, unlike for NA extractions from tissue, RNA carrier was added to the samples for nucleic acid (NA) extraction from cell culture. To mimic the NA obtained from infected tissue, a volume of 150 μL of ground larvae (1:10 w/v) was mixed with 50 μL of cell culture supernatant infected with the selected virus.

### 2.3. RT-PCR Amplification for Sequencing

**Figure 1:**
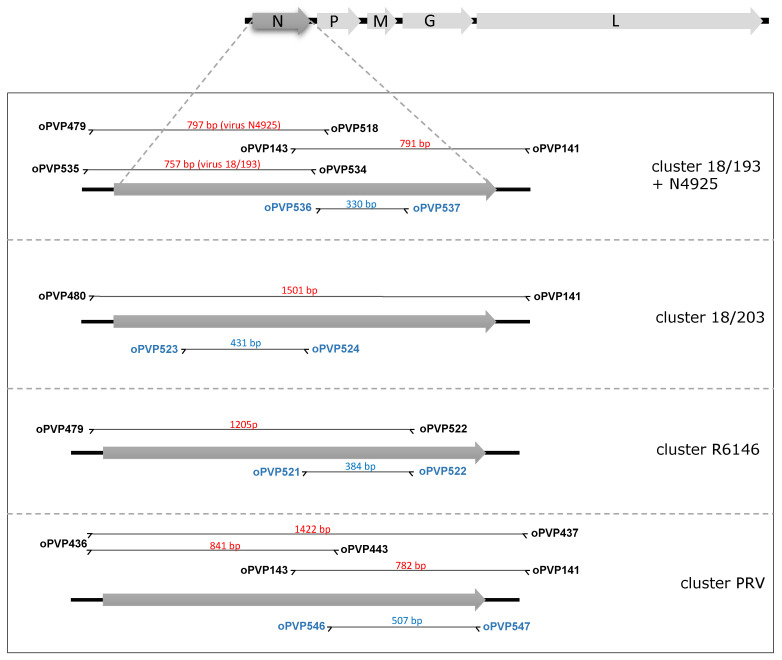
Positions of the primers used for amplifying genomic portions of the nucleoprotein (N) ORFs of percid perhabdoviruses. A typical perhabdovirus genome, with its five canonical genes, is shown at the top. For the initial identification of the viruses, different sets of primers have been used depending on the virus or depending on the sequences already available. Primers designed for detecting all isolates belonging to a specific cluster are in blue. The size of each amplicon is indicated.

For some viral isolates, a partial sequence of the N ORF was already available [3]; therefore, only the missing portion was amplified to construct a complete sequence (Figure 1). For other isolates, the complete ORF was obtained using PCRs amplifying either in a single fragment or in two overlapping fragments. The amplicons were produced using primers adapted to each genogroup based on previously obtained sequences of the N or G gene. For all the RT-PCR reactions, a SuperScript III One-Step with Platinum Taq High-Fidelity kit (Invitrogen, Carlsbad, USA) was used according to the manufacturer’s protocol, by adapting the polymerization time to the length of the expected product and the melting temperature of the primers. Before sequencing, PCR products were purified (Macherey-Nagel) and TA-cloned in a PCR4-TOPO vector (Invitrogen). For each amplicon, three clones were sequenced in both orientations with universal primers using the Sanger method and a 3130 Genetic Analyzer (Applied Biosystems, Foster city, USA).

### 2.4. RT-PCR Detection

All the available sequences of the N ORFs were aligned, and specific sets of primers were manually designed in regions that were fully conserved within a genogroup but showed substitutions in the three other groups. Four pairs of genogroup-adapted primers were designed: oPVP521 and 522, oPVP523 and 524, oPVP536 and 537, and oPVP546 and 547 (Figure 1 and Table S2). They were used at 0.4 μM in a final reaction volume of 50 μL containing 2 μL of total RNA. For RT-PCRs, a SuperScript III One-Step with Platinum Taq High-Fidelity kit was used, starting with a reverse-transcription step of 30 min at 58 °C, followed by 2 min at 94 °C and 40 cycles including 30 s at 94 °C, 30 s at 60 °C and 30 s at 68 °C. After PCR, a volume of 10 μL of these reactions was loaded on a 2% agarose E-Gel (Invitrogen) and migrated for 15 min before observation under UV light. 

### 2.5. Next-Generation Sequencing

To obtain the complete viral genomes, next-generation sequencing (NGS) was performed for five selected samples (18/193, 18/203, P8350, R6146 and 16/65) based on Illumina sequencing technology as previously described [18,19]. First, RNA extraction was performed on 200 μL virus-infected cell culture supernatants using Direct-zol RNA MiniPrep kit (Zymo research, Irvine, USA) according to the manufacturer’s recommendations, with a 50 μL final volume of elution. cDNA synthesis was performed on approximatively 50 ng of RNA (8 μL) with random hexamers (Invitrogen) and Superscript^TM^ III reverse transcriptase (Invitrogen) according to the manufacturer’s instructions. Afterward, double-stranded (ds) DNA was synthesized in an 80 μL reaction mixture incubated at 16 °C for 2 h for each viral cDNA. This mixture included 20 μL of fresh cDNA, 10x Second-Strand Synthesis Reaction Buffer (New England Biolabs), 3 μL of 10 mM dNTP mix (Invitrogen), 1 μL (10 U) of *E. coli* DNA ligase, 4 μL (40 U) of *E. coli* DNA polymerase I, 1 μL (5 U) of *E. coli* RNase H (New England Biolabs, Evry, France) and 43 μL of nuclease-free water. Finally, the dsDNA was purified using AMPure XP (Beckman Coulter, Villepinte, France). dsDNA libraries were constructed using the Nextera XT kit (Illumina, Evry, France) and sequenced using a 2 x 150 nucleotide paired-end strategy on the NextSeq500 platform. The NGS data were analyzed on the Pasteur Galaxy platform [18,19,20]. The viral sequence contigs were assembled and manually edited to produce the final sequences of the viral genomes using Sequencher 5.2.4 (Gene Codes Corporation, Ann Arbor, USA). The quality and accuracy of the final genome sequences were checked with a final mapping of the reads obtained from NGS and visualized using Tablet [21]. The 5′ ends of the viral genomes were confirmed by rapid amplification of cDNA ends (RACE) using the SMARTer RACE 5′/3′ kit (TaKaRa, Saint-Germain-en-Faye, France). The 3′ ends were phosphorylated and ligated using T4 RNA ligase (TaKaRa) then amplified using hemi-nested PCR as previously described [22]. Complete genome sequences, as well as N and G ORF sequences, were deposited in GenBank (Table S1).

### 2.6. Phylogenetic Analysis

DNA sequences obtained from Sanger sequencing were assembled and the consensus sequence edited using VectorNTI11.5 (Invitrogen). The sequences were aligned with the ClustalW function of MEGA7 [23]. Maximum-likelihood (ML) analyses were performed using a transition/transversion ratio of 2.0 with empirical base frequencies and the HKY85 substitution model implemented in the MEGA7 program [23]. Bootstrap analyses were performed using 1000 replications. Values greater than 90% were considered as strong evidence for robust phylogenetic groupings.

## 3. Results

### 3.1. Study of the N Gene

**Figure 2:**
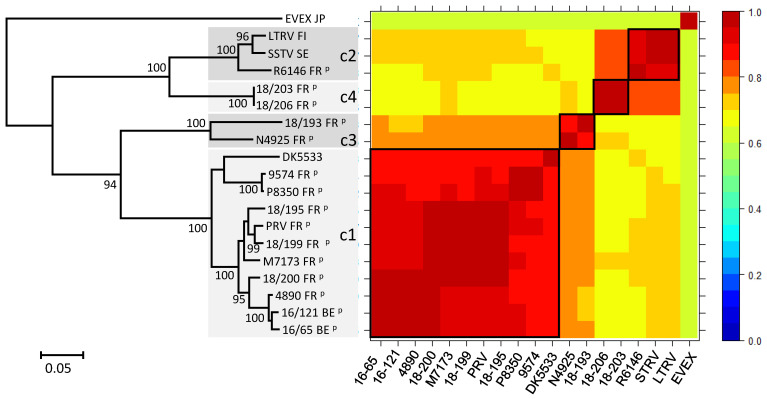
Phylogenetic analysis of the nucleoprotein ORF and identity levels between viruses. In the maximum-likelihood phylogenetic tree, the country of origin (two-letters code) is indicated after the name of the isolate and the P superscript indicates isolation from a percid fish. In the similarity matrix, similarities are color-coded. The black frames and the shaded blocks arbitrarily delimitate four clusters (c1–c4). The eel virus European X (EVEX) (GenBank FN557213) was used as an outgroup.

A set of 10 viruses isolated from percids in France was genetically identified using the complete N ORF sequence, newly obtained or completed from partial sequences previously published. They were aligned with eight other *Perhabdovirus* sequences available in GenBank and with a sequence of EVEX. All the full-length N ORFs shared genetic similarities with other percid perhabdoviruses, although at different levels (Figure 2). The new sequences exhibited 68.4 to 98.5% of nucleotide identity with other isolates belonging to *Perch perhabdovirus* and *Sea trout perhabdovirus*. Four main clusters were distinguished: (i) a main cluster (c1) of sequences related to PRV, which can be divided into subgroups previously named A, B and C; (ii) a second cluster (c2) that groups three viruses, LTRV, SSTV (previously denoted as genotype F) and R6146 (previously denoted as genotype E); (iii) a cluster (c3) that includes isolates 18/193 and N4925, the latter described elsewhere as genotype D; and (iv) a fourth new cluster (c4) including two identical sequences (isolates 18/203 and 18/206). These two isolates have nucleotide identities between 80 and 85% compared with the members of the c2 (SSTV) group. Therefore, whether these two latter clusters form a unique genogroup or not depends on the defined threshold level of similarities between two groups of sequences, i.e., at 80 or 85%. A phylogenetic tree of the translated products of the N ORF gave a pattern very similar to that of the nucleotide sequences, although the two clusters containing 18/203 and SSTV were more strongly genetically linked and formed one main cluster (Figure S1). 

Interestingly, genetic diversity was observed among several isolates originating from the same fish farm. For instance, three very distinct viruses—namely R6146, 18/199 and 18/200—were isolated from the same site that had regularly introduced fish captured in the wild. Isolates 18/199 and 18/200, both in the PRV cluster, were isolated in 2001, but they share only 95% of nucleotide identity (Figure 2). Unfortunately, the data concerning the initial origin of the fish before introduction to the farm have been lost. However, it is known that the individual fish carrying isolates 18/195 and PRV, both very similar to 18/199, originated from Lake Annecy (located in the French Alps) and the Solognes region (central France), respectively. Thus, it is possible that 18/199 was imported from one of these two regions into the farm. Regarding virus R6146, isolated 4 years later in 2005, it belonged to the SSTV cluster and was therefore very different from 18/199 and 18/200. Conversely, two nearly identical viruses, 9574 and 18/212, were found in two distinct sites, strongly suggesting the dissemination of the same virus to different farms.

### 3.2. Complete Genome Sequences

**Figure 3:**
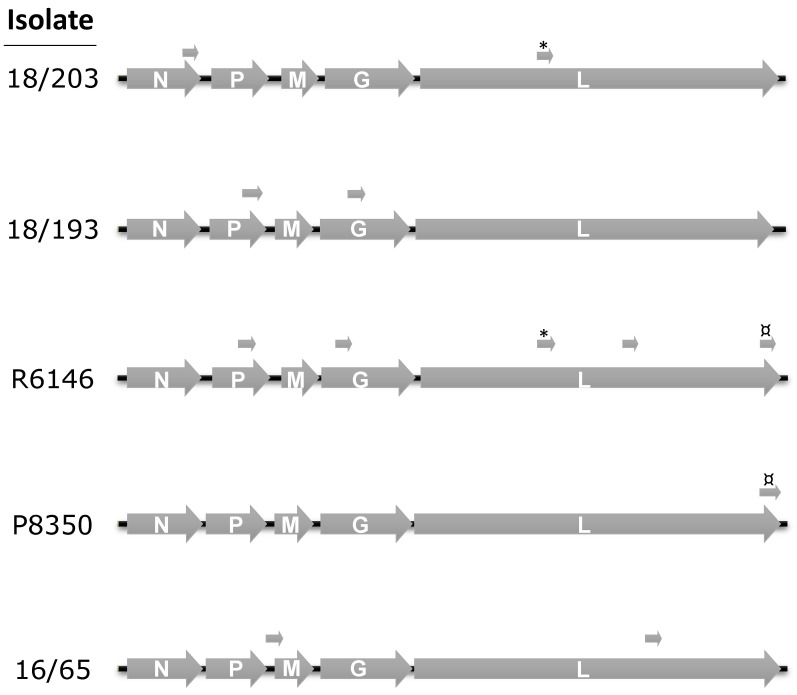
Genetic maps of the five newly sequenced genomes. Arrows above the maps indicate short ORFS (>180 nt; see text). Symbols ¤ and * indicate pairs of short ORFs with minimal homology.

At least one virus representing each of the four genetic clusters was further analyzed using whole genome sequencing. However, within the large cluster containing PRV, two genetically related viruses (16/65 and P8350) were sequenced because—based on the N gene—they showed some divergence between one another and from PRV. In total, five complete sequences of genomes were newly obtained (Figure 3). At their 3′ ends, their leader sequences included the same 13 nt and the 5′ trailer sequences shared the same 20 nt, which contained the complementary inverse of the first 13 nt. In particular, all five genomes had the same two nucleotides (AC) at the 3′ end and had the corresponding inverse dinucleotide (GT) at the 5′ end, although these were missing from the previously published PRV genome (as reported in [13]). All five genomes carried the five canonical genes N, P, M, G and L present in rhabdoviruses. The genomes varied from 11,438 to 11,619 nt, three genomes being longer (by about 175 nt) than the two others (namely 16/65 and P8350). The differences in size were concentrated in the untranslated intergenic regions of N-P, P-M and G-L at the 5′ end of the genome and in the P ORF. A number of non-canonical small ORFs with sizes greater than 180 nt were detected, as for other members of the *Rhabdoviridae* [24]. Some had no homology at all with any protein in a BLASTx search in the NCBI database, whereas others had low similarities with very short portions of proteins with no identified function. In one case, a short translated ORF of isolate R6146 exhibited 27% identity with a portion of a chloride channel protein 1-like of a fish (*Sphaeramia orbicularis*). This ORF was also present in isolate 18/203, sharing 57% amino-acid similarity with the R6146 ORF, but only 17% with the same partial fish protein. In particular, the sequence QQxGHxGxYR was conserved in the three translated ORFs. In another case, the amino-acid sequence of a short ORF in isolate R6146 exhibited low similarity (37%) with about two-thirds of a short ORF in isolate P8350. No hypothetical function was identified for the products of either putative ORF.

Isolate 16/65, obtained from moribund pike-perch larvae in Belgium in 2016, is the most recent in the present study and has already been partially sequenced [8]. Its complete size is 11,438 nt, which makes it the shortest genome among the five new sequences reported in this study. It encodes the same five genes as the published PRV genome, with which it shares the highest identity (94%), as previously demonstrated based on the N and G genes. Strikingly, it differs strongly in a narrow portion of the L gene, with a series of substitutions and apparent insertions in the published PRV genome. To solve this uncertainty, we amplified this region and sequenced it (using the Sanger method), starting from an isolate of PRV maintained in our laboratory, as well as from two other highly related viruses (9574 and 18/199). In particular, the present PRV sequences differed from the published one, with the latter exhibiting abnormal insertions not observed in the four other related viruses (PRV of this work, 18/199, 9574 and 16/65) (Figure S2), suggesting inaccurate sequencing or genetic changes in this region for the previous published full-length PRV. The genome of 16/65 has two small ORFs, one in the intergenic P-M region and one within the L gene, but neither ORF had a putative transcription signal located upstream.

Isolate P8350 was isolated from perch in France in 2004. In a previous study, it was shown to belong to genogroup B of PRV, sharing only 90% of nucleotide identity in the G ORF. Its complete genome is 11,443 nt and shares 89% identity with the published sequence of PRV (including the divergent region) and 88% identity with 16/65. This genome had a single short ORF at the end of the L ORF, with potential transcription signals relatively far upstream (>100 nt).

**Figure 4:**
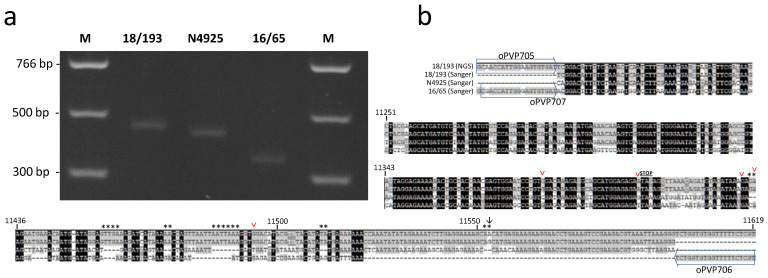
Amplification and sequencing of the 5′-end of the genome of three selected isolates (18/193, N4925 and 16/65). (**a**). Electrophoresis of the amplicons after specific amplification of three isolates using the oPVP705 and oPVP706 primers (viruses 18/193 and N4925) or oPVP707 and oPVP706 primers virus 16/65). M, PCR marker (NEB). (**b**). Alignment of the sequences obtained after cloning amplicons and Sanger sequencing. The position of the PCR primers and their sequences are indicated in the alignment. The stop codon of the RNA polymerase (l) gene is indicated. Asterisks indicate the deletions in N4925 compared to 18/193. The sequence obtained by NGS for isolate 18/193 is added for comparison with one obtained by Sanger sequencing of a particular plasmid clone of the same virus; there is only one difference (↓). However, in the amplified region, five additional sites (∨) were variable (A/G) between three distinct clones of 18/193. These ambiguities were also found in the Illumina reads (not shown) and reflect intra-isolate genetic diversity.

Isolate 18/193 was the oldest of our collection and was hereby studied for the first time. It was isolated in 1999 from symptomatic wild young perch collected in Lake Leman. The newly obtained sequences of its N and G genes clearly showed that it is related to, though distinct from, isolate N4925 isolated in 2003 from perch, sharing 88 and 85% identity in the N and G ORFs, respectively. Its genome size is 11,619 nt and is the longest of the five new genomes. An additional ATG is present upstream from the conserved start codon of the N gene, with a potential addition of 44 amino acids to the N protein. However, no canonical transcription signal was observed upstream of this first start sequence, except a potential AACAG signal—though at a distance of 88 nt—and another possible signal (AAGAG), which was closer, at only 38 nt upstream. The AACAG motif was present in the other canonical genes of 18/193 and in all the genes of the other percid perhabdoviruses. For the M gene, an ATG was also observed upstream of the conserved start sequence but, again, without any apparent transcription signal upstream. The 18/193 isolate shared only 74% and 73% identities with the nearest complete sequences, those of P8350 and 16/65, respectively. Surprisingly, a 78 nt sequence present near the 5′ end of the genome was absent in the four other genomes. This insertion was confirmed by amplifying and Sanger-sequencing a 438 bp fragment at the 5′ end of the genome (Figure 4). To test if this insertion was specific to that isolate or specific to the genogroup to which it belongs, the same region was amplified from the closely related isolate N4925. Again, a similar, but slightly shorter, insertion was found, differing by a few substitutions compared with 18/193 (Figure 4a). The difference in size is due to the absence of a total of 19 nt, dispersed over six very short deletions (Figure 4b). Comparatively, electrophoretic analysis of the similar PCR amplicon obtained with isolate 16/65 (related to PRV) showed a much shorter size, as expected. We therefore concluded that the presence near the end of the genome of an additional sequence of unknown function is specific to the genogroup including 18/193 and 18/211. Two small ORFs greater than 180 nt were identified in this isolate 18/193, both included within a canonical ORF, either P or G. The first ORF had a potential transcription AAGAG motif 40 nt upstream of its ATG signal, and the second ORF had an AACAG motif 96 nt upstream the ATG signal. 

The virus R6146 was isolated in France in 2005 from perch larvae. In a previous study, it was shown that the sequences of its complete G ORF and partial N ORF had more similarities with SSTV than with PRV. Therefore, R6146 was included in genogroup F. The full-length genome of R6146 (11,602 nt) confirmed this relationship, sharing 91% identity with SSTV and LTRV over nearly 5.9 kb. R6146 is therefore the first complete genome representing the *Sea trout perhabdovirus* species. It shared only 68% of nucleotide identity with PRV, which is the prototype of *Perch perhabdovirus* species. Among the new genomes, R6146 had the highest number of short putative ORFs, and all five were found in the canonical ORFs: one in P, one in G and three in L. Only two, located in the L gene, had an AACAG motif upstream the ATG signal, at distances of 63 or 93 nt.

The virus 18/203 represents a new genogroup. Its genome is 11,610 nt. This virus shares 68% identity with the published PRV genome and 79% with R6146. A series of similar insertions and deletions in the P gene revealed the genetic proximity of 18/203 to R6146, compared to the four other full-length sequences (18/193, P8350, 16/50 and PRV). This virus had two short ORFs, one in the N gene with an AAGAG motif 53 nt upstream of the ATG signal and the other in the L gene without any apparent transcription initiation motif.

**Figure 5:**
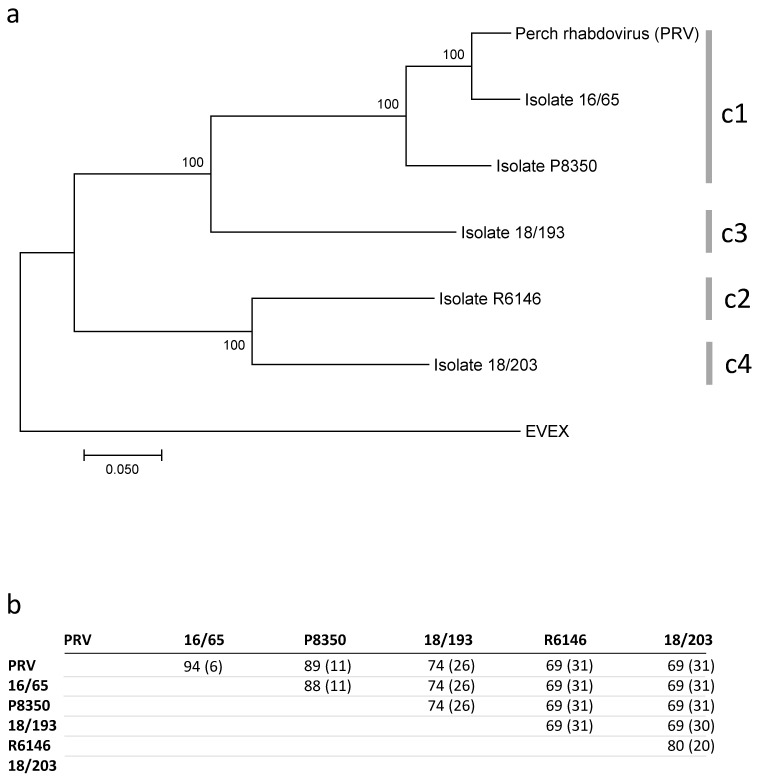
Genetic relationships between concatenated ORFs of percid perhabdoviruses. **a**. Maximum-likelihood phylogeny with 1000 bootstraps; eel virus European X (EVEX, Genbank FN557213) was used as an outgroup. Corresponding clusters (c1-c4) defined with the N ORFs are indicated by the vertical traits. **b**. Levels (%) of nucleic acid identities (and divergence in brackets) of the contatenated ORFs.

The five new full-length sequences were compared with one another and with PRV by aligning the concatenated ORFs, using EVEX as an outgroup. On the phylogenetic tree, two strongly supported monophyletic clades were observed, each carrying two branches (subclades) (Figure 5). In one clade, one branch grouped PRV, 16/65 and P8350, differing by 5 to 12% in identity. The second branch carried isolate 18/193, which exhibited only 74% identity with members of the previous clade including PRV. The second clade carried two divergent viruses, R6146 and 18/203, both differing by 20% in their nucleotide sequences. Strikingly, isolate 18/193, which was genetically included in the PRV clade, had a genome size closer to the genome sizes of the isolates of the second clade. 

The six complete L ORFs (five new + PRV) diverged by 5 to 29% at the nucleotide level. Importantly, the levels of divergence varied from 19 to 29% between representatives of the four main genetic branches observed using the concatenated genes (Figure 5). Not surprisingly, a phylogenetic tree constructed with only the translated products of the L gene gave a topology very similar to the one obtained with the concatenated genomes (Figure S1). Two other genes often used for the identification of perhabdoviruses, namely N and G, exhibited roughly similar levels of relatedness compared with L (Table S3). The N nucleic and amino-acid identity levels varied from 70 to 82% and from 71 to 93%, respectively, between representatives of the four main identified clusters. For the G ORF, the nucleic and amino-acid identity varied from 66 to 78% and 68 to 88%, respectively.

### 3.3. PCR Detection

**Figure 6:**
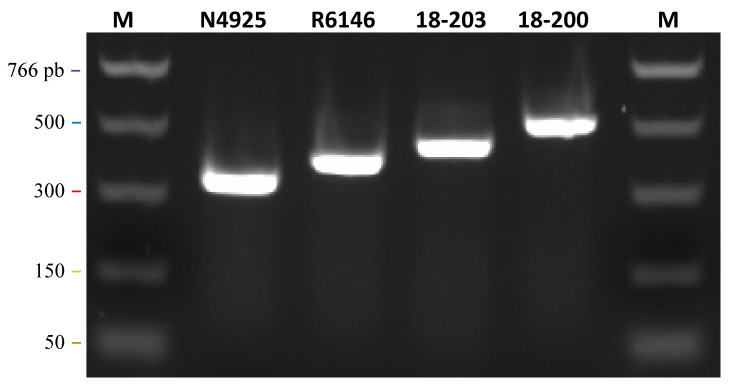
Amplification of distinct viruses with different primer sets. Representatives of each of the main genogroups were amplified during the same PCR run, changing only the set of primers: N4925 (primers oPVP536 and oPVP537), R6146 (oPVP521-522), 18/203 (oPVP523-524) and 18/200 (oPVP546-547). Expected sizes: 330 bp (isolate N4925), 384 bp (R6146), 431 bp (18/203) and 507 (18/200). M, PCR size marker (NEB).

For members of the three clusters c2, c3 and c4, no specific PCR assay had been set up to date. In an attempt to design PCRs adapted to the amplification and sequencing of portions of viruses from all the clusters, we selected sets of discriminant primers based on conserved regions in the N ORF but as divergent as possible from sequences of the other clusters. We first verified that each primer set effectively detected a representative of the corresponding cluster. Four conventional PCR (cPCR) assays, run simultaneously and differing only by the primers and the RNA sample, produced signals at the expected sizes for each isolate (N4925, R6146, 18-203 and PRV) (Figure 6).

**Figure 7:**
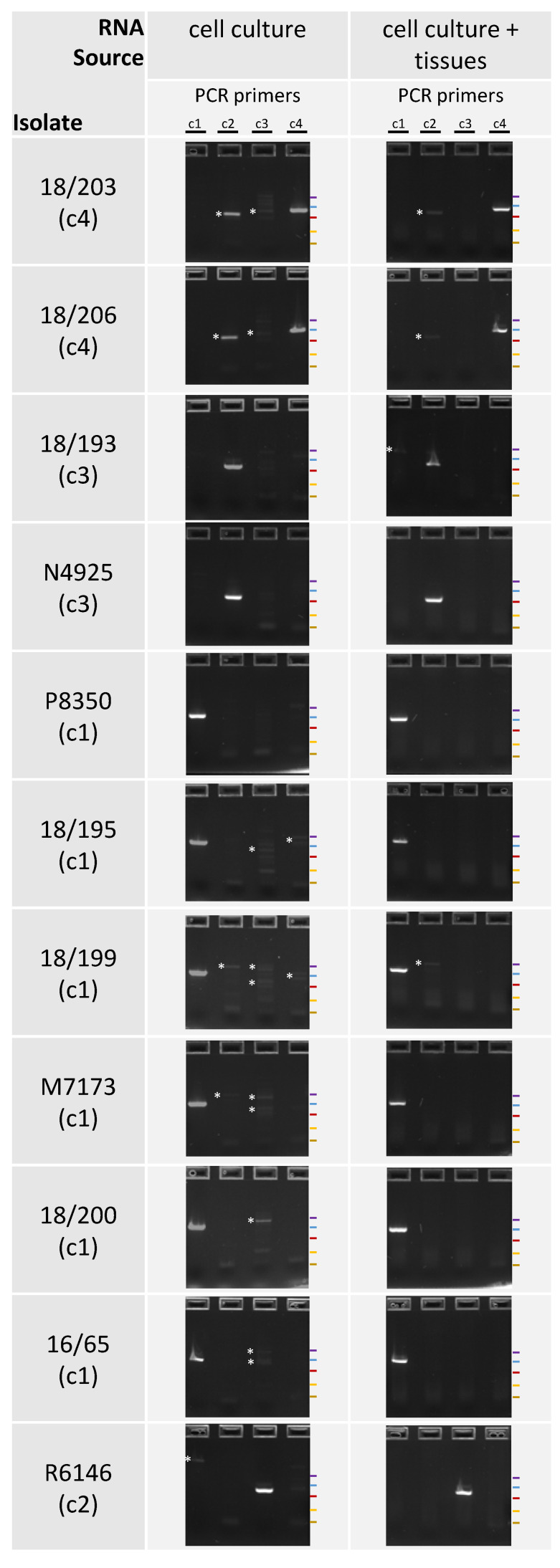

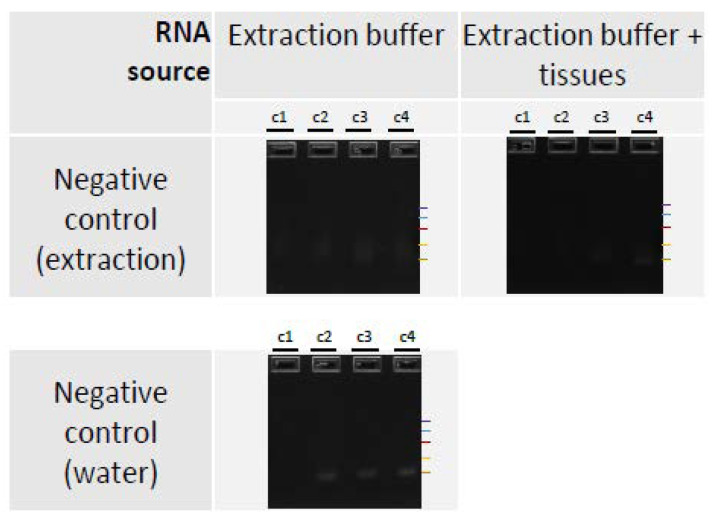
Results of conventional PCRs performed with a series of viral isolates. Four pairs of primers were used for the amplifications, each one adapted to a specific cluster: c1, c2, c3 and c4. For each virus, the results are given for the NA extracted just from cell culture (left) or by mixing with healthy fish tissues (right). In brackets, is indicated the genetic cluster of each virus. The colored bands indicate the positions of the bands of the molecular ladder (NEB): 766 bp, 500 bp, 300 bp, 150 bp, 50 bp. The asterisks indicate non-specific products.

The specificity of each set of primers was then tested using 11 viruses belonging to the four clusters, starting with nucleic acids extracted from infected cell cultures, mixed (MNA) or not mixed (NMNA) with healthy fish tissues. With MNA and NMNA, each of the four PCRs produced a clear signal at the expected size for the cognate virus and no signal most of the time for the three heterologous viruses (Figure 7). However, weak non-specific products were sometimes observed for the heterologous viruses, manifested as faint bands of sizes different from the expected size. These non-specific products were observed more frequently starting in the NMNA samples than in the MNA samples. The sequencing of these occasional non-specific products revealed that they consisted of non-targeted portions of the viral genomes, and not non-specific amplicons from the host. It was concluded that the sets of primers can amplify genomic portions of viruses of their cognate genogroup, facilitating accurate genetic identification, but are not specific enough for a diagnostic assay. 

## 4. Discussion

Despite the strong need to elucidate and trace the viral outbreaks that have been occurring in various European aquaculture farms of freshwater fish species for several decades, the molecular detection and the classification of percid perhabdoviruses has been limited. Classification based on the host range seems impossible because the biological properties of these viruses are usually poorly or not at all studied. Moreover, some of these viruses are probably prone to host switching. For instance, within the species *Perch perhabdovirus*, highly genetically related viruses have been found in percids and non-percid species such as brown trout (*Salmo trutta*) and grayling (*Thymallus thymallus*) [2]. Similarly, in the *Sea trout perhabdovirus* species, two nearly identical viruses have been identified in perch and black-bass (*Micropterus salmoides*) [3]. Therefore, genomic sequences are undoubtedly of high interest for classifying these viruses. Unfortunately, only limited genetic data are available for perhabdoviruses. The ICTV has focused only on the partial L gene for species demarcation within the genus, noting divergences of 17–21% between members of the species *Sea trout perhabdovirus* and *Perch perhabdovirus*. By comparison, within the genus *Novirhabdovirus*, a viral genus specific to fish, members of the four assigned viral species show a minimum of 31% of divergence in the G gene (ICTV). For perhabdoviruses, the two species demarcation criteria are a minimum nucleotide sequence divergence of 15 % in L genes and distinct antigenic patterns (talk.ictvonline.org, March 2020). However, regarding the latter criterion, antisera are still little available and little characterized. Moreover, puzzling and sometimes contradictory results have been obtained in the published serological studies. For instance, the immunofluorescence results of one study showed that the DK5533 isolate is strongly recognized by a serum raised against PRV but another study failed to demonstrate this antigen recognition [5,7]. Another argument against the use of antigenic data for classification is the apparent difference in serological patterns between two viruses exhibiting as high as 99.2 % identity in the G protein [5]. Finally, a weak, nevertheless existent, cross-reaction has been observed in immunofluorescence tests between a PRV antiserum and a very divergent representative of *Sea trout perhabdovirus*, namely LTRV. It is thus difficult to rely on serological tests to group viruses in the same cluster or to distinguish viruses of different clusters. Clearly, partial and complete genomic sequences are key elements for assigning perhabdoviruses to species.

However, only one complete percid perhabdovirus genome has been available to date (PRV) and only about half of the genomes of two SSTV isolates from Northern Europe had been sequenced [13,15,16]. Another issue is the molecular detection and identification of any new isolate. With that goal, the largest possible library of sequences targeting a gene of special interest would be useful. In a preliminary step, we genetically characterized the present virus collection using the complete N gene. For the family *Rhabdoviridae*, this gene is a marker of interest, because it is a reliable indicator of genetic diversity and is a target for PCR detection, being allegedly more expressed than the other genes.

In a previous study, phylogenies on the complete G ORF and the partial N ORF proved to be highly consistent [3]. The present panel of 10 new complete N sequences, together with the eight others available in GenBank, also gave phylogenies consistent with the previous ones. However, the additional sequences further increased the genetic diversity described within the genus and were useful to reconsider the relationships between the viruses and to refine the classification. We identified four main clades (genogroups). For three reasons, this separation into four genogroups appears more appropriate than the one proposed a decade ago based on eight groups (A to H). First, groups A, B and C appear to be more related than previously considered and are likely part of the same cluster, c1, represented by PRV. Secondly, the two genogroups E and F are also more similar than previously observed and can be merged into a single cluster, c2. Thirdly, viruses in genogroups G (EVEX) and H (Siniperca chuatsi rhabdovirus) display strong divergence with A-F, confirming their assignments to separate species, clearly distinct from *Perch perhabdovirus* and *Sea trout perhabdovirus*. In our study, the most represented group was the cluster c1, which was further divided into two sub-clusters with viruses of distinct geographic origins and different hosts. A second cluster (c3) was composed of the pair of isolates N4925 and 18/193. A third cluster (c2) consisted of a series of viruses related to SSTV. The fourth cluster (c4), never described before, contained two almost identical viruses (18/203 and 18/206) isolated in France in 2002. 

We sequenced de novo five selected complete perhabdovirus genomes representing the four clusters c1–c4. With this work, a complete genome of a member of the *Sea trout perhabdovirus* species is now available. It shared 68% of identity with each of the three fully sequenced representatives of the PRV group. In addition, two other members of the PRV cluster were also fully sequenced, as well as two other viruses distinct from the other viruses according to their N gene divergence. The sequences of three viruses were slightly longer than the two others. The sizes of the viruses were only partially associated with the genogroup: the two PRV-related viruses were shorter than the two SSTV-related viruses; however, virus 18/193 had a size more similar to the SSTV group although it was genetically more related to PRV. This longer size is partially due to an apparent 78 nt insertion at the end of the genome, not found either in the four other more recently isolated genomes or in the reference PRV strain which is older. Interestingly, a similar insertion was found in another strain (N4925) related to 18/193. Therefore, such variations are probably linked to the molecular evolution of this genogroup. It will be interesting in the future to monitor the evolution of this region in all the related strains. 

Among the five genomes, in addition to the canonical genes, a dozen putative short ORFs greater than 180 nt in size were detected in total. Most were not conserved among the five viral genomes. Only two ORFs, both in the L gene, showed one homologous ORF in another viral genome. For none of the putative products of these ORFs was a function deduced from their primary amino-acid sequence. Some had a potential transcription signal at a reasonable distance upstream from the start codon, and others did not. Therefore, whether these ORFs are, or not, expressed and functional is unknown. A model for the evolution of accessory genes in the *Rhabdoviridae* involves the emergence of such genes starting from small ORFs created de novo after mutations [24]. If expressed, these ORFs may affect the molecular biology of the viruses by modifying the expression of the main genes that carry them. An example of the potential importance of such ORFs is the C′ protein of vesicular stomatitis virus (VSV), which is encoded by the P gene and has a strong positive effect on the polymerase activity, by enhancing both the level and fidelity of mRNA synthesis [25].

Without available serological data, the new sequences provide key data that help to classify the percid perhabdoviruses either based on their complete L gene, as proposed by the ICTV, or based on longer sequences such as the concatenated genes. Two viruses with the L ORF or concatenated genes with less than 85% nucleic acid identity can be considered as different species. By using these classification criteria, the viruses PRV, R6146 (and SSTV), N4925 and 18-203 can be reassigned to four species, including two new ones with *Perch perhabdovirus 1* (c1, representative *PRV*), *Perch perhabdovirus 2* (c3, with N4925), *Sea trout perhabdovirus 1* (c2, with R6146) and *Sea trout perhabdovirus 2* (c4, with 18-203). The names consider the existence of relative genetic proximities between some pairs of viruses, for instance PRV with N4925 on one hand, and R6146 with 18-203 on the other hand. In an alternative classification, simpler and based only on the history of the discoveries of the first representatives of each group, the names *Perch perhabdovirus* 1–4 would be used, but such a nomenclature would have the strong drawbacks of eliminating the name *Sea trout perhabdovirus*, already recognized by the ICTV, and also ignore the genetic relations between the species.

The high genetic diversity of percid rhabdoviruses in France is intriguing. The various clusters did not show any clear correlation with the geographic origins of the studied viruses or the host. For instance, viruses R6146, 18/200 and 18/199 were genetically different but were found in the same site, suggesting separate introductions of contaminated fish. We also found two almost identical viruses in different farms without any waterway connections, strongly suggesting fish movements between regions due to trade. Except for a few viruses originating from the wild (ponds and Alpine lakes), most were isolated from farms that had introduced fish directly from the wild or from other farms. Therefore, the high diversity is probably due to the different geographical origins of the viruses. There are thousands of lakes, ponds and rivers in France, which may host viruses that have diverged over time and that were accidentally introduced to farms via broodstock or eggs. Moreover, trade has undeniably instigated numerous exchanges of fish between France and other European countries. Therefore, the reservoir of viruses is much larger than just French freshwaters and can be extended to all of Europe, including its marine waters, with some viruses being found in seawater or lakes in connection with seawater [7,16]. As an example of the massive circulation of fish within mainland Europe, pike-perch were repeatedly introduced in Denmark over the last 200 years from wild fish originating from Germany and Sweden [26]. The probable transfer of viruses between sites and regions is illustrated by several examples in different genogroups. In France, three highly related viruses from the same genogroup were isolated from three distinct sites: Lake Annecy in the Alps (18/195), a pond in Sologne (PRV) and a non-commercial farm that captures fish from Alpine lakes (18/199). In mainland Europe, the perfect identity between two viruses from France (16/179 and 16/183) and two viruses from Belgium (16/121 and 16/122) was previously hypothesized to be the common origin of infected fish, probably a third country [8]. The cross-species transmission and regional movements of fish also explain why viruses related to SSTV have been found in France and in Finland in perch and lake trout, respectively.

Each virus of the present collection was readily detected using newly designed cPCR tests, producing amplicons of group-specific sizes. In a previous study, a specific cPCR assay had already been set up for the PRV group; however, it occasionally showed some cross-reactions with the other genogroups () [8]. Therefore, we developed new primers targeting this same group here. For the three other genogroups, these are the first tools available, starting from nucleic acids extracted from infected cell cultures and mixed, or not, with RNA from healthy fish tissue. It must be emphasized that, even with the stringent conditions used here, this detection tool was not perfectly specific, with occasional cross-reactions with other related viruses. This insufficient specificity requires sequencing the PCR product to confirm its identity. Further work is needed to develop virus- or genogroup-specific diagnostic tools with higher specificities, based on real-time PCR for instance. Nevertheless, when clearly positive, the detection test developed here produces enough genetic data for a preliminary identification based on a portion of the N gene. More accurate identification is possible by sequencing the complete N gene using other sets of primers, as demonstrated.

## 5. Conclusions

A comprehensive overview of the diversity of the perhabdoviruses is needed to prevent their continued spread. Our genetic data and new amplification methods can contribute to the rapid and accurate identification of any new isolate highly related to those already characterized, which is crucial for tracing outbreaks. Due to the global lack of active surveillance in Europe, it is likely that known and yet-unknown genotypes will emerge in the next decade. Furthermore, given the host-switching capacity of these viruses and their frequent geographic translocations, the emergence of any of these viruses will likely affect various hosts, not only those already recognized (percids and non-percids), but also possibly new freshwater hosts that are increasingly farmed worldwide, such as tilapias. 

## Supplementary Materials

The following are available online at https://www.mdpi.com/1999-4915/12/6/649/s1, Figure S1: Phylogenetic trees of the predicted proteins encoded by the nucleoprotein (N) and RNA polymerase (L) genes. Figure S2: Comparison of portions of the RNA polymerase (*l*) gene of related viruses. Table S1: Viral sequences studied in the present work. Table S2: List of the primers used in this study. Table S3: Identities levels of three selected ORFs of the five newly obtained viral genomes.
