# Supplementary material for: Revisiting the Classification of Percid Perhabdoviruses Using New Full-Length Genomes
Source: Viruses. 2020 Jun 16;12(6):649. doi: 10.3390/v12060649 (PMC7354598; doi:10.3390/v12060649)
Supplement: Supplementary file 1 [file viruses-12-00649-s001.zip › Pallandre figure S1 revised.pptx]

## Slide 1
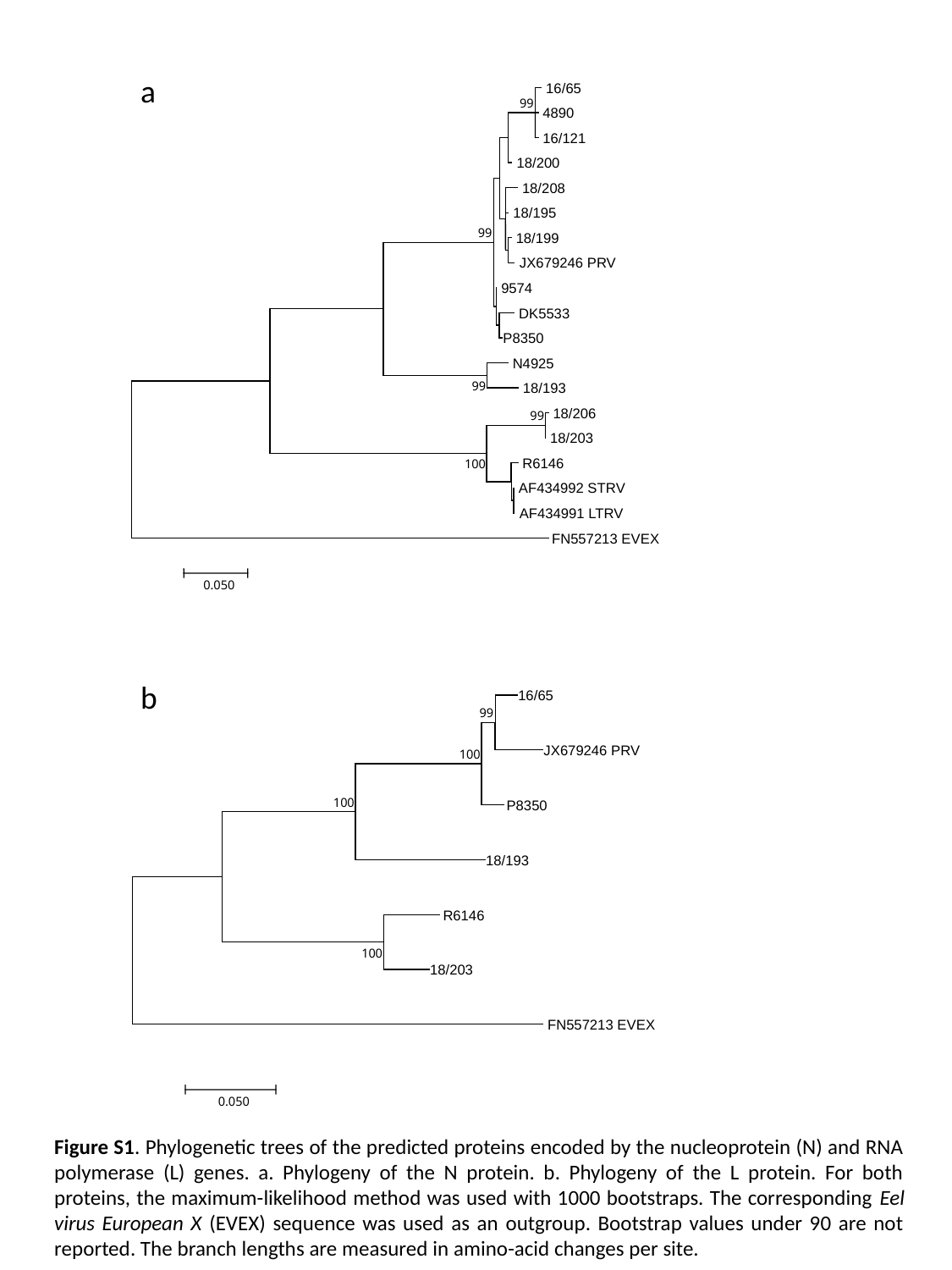

a
 16/65
99
 4890
 16/121
 18/200
 18/208
 18/195
99
 18/199
 JX679246 PRV
 9574
 DK5533
P8350
 N4925
99
 18/193
 18/206
99
 18/203
 R6146
100
 AF434992 STRV
 AF434991 LTRV
FN557213 EVEX
0.050
16/65
99
JX679246 PRV
100
100
P8350
18/193
R6146
100
18/203
 FN557213 EVEX
0.050
Figure S1. Phylogenetic trees of the predicted proteins encoded by the nucleoprotein (N) and RNA polymerase (L) genes. a. Phylogeny of the N protein. b. Phylogeny of the L protein. For both proteins, the maximum-likelihood method was used with 1000 bootstraps. The corresponding Eel virus European X (EVEX) sequence was used as an outgroup. Bootstrap values under 90 are not reported. The branch lengths are measured in amino-acid changes per site.
b
