# Supplementary material for: Revisiting the Classification of Percid Perhabdoviruses Using New Full-Length Genomes
Source: Viruses. 2020 Jun 16;12(6):649. doi: 10.3390/v12060649 (PMC7354598; doi:10.3390/v12060649)
Supplement: Supplementary file 1 [file viruses-12-00649-s001.zip › pallandre Figure S2 gene L insertions revised.pptx]

## Slide 1
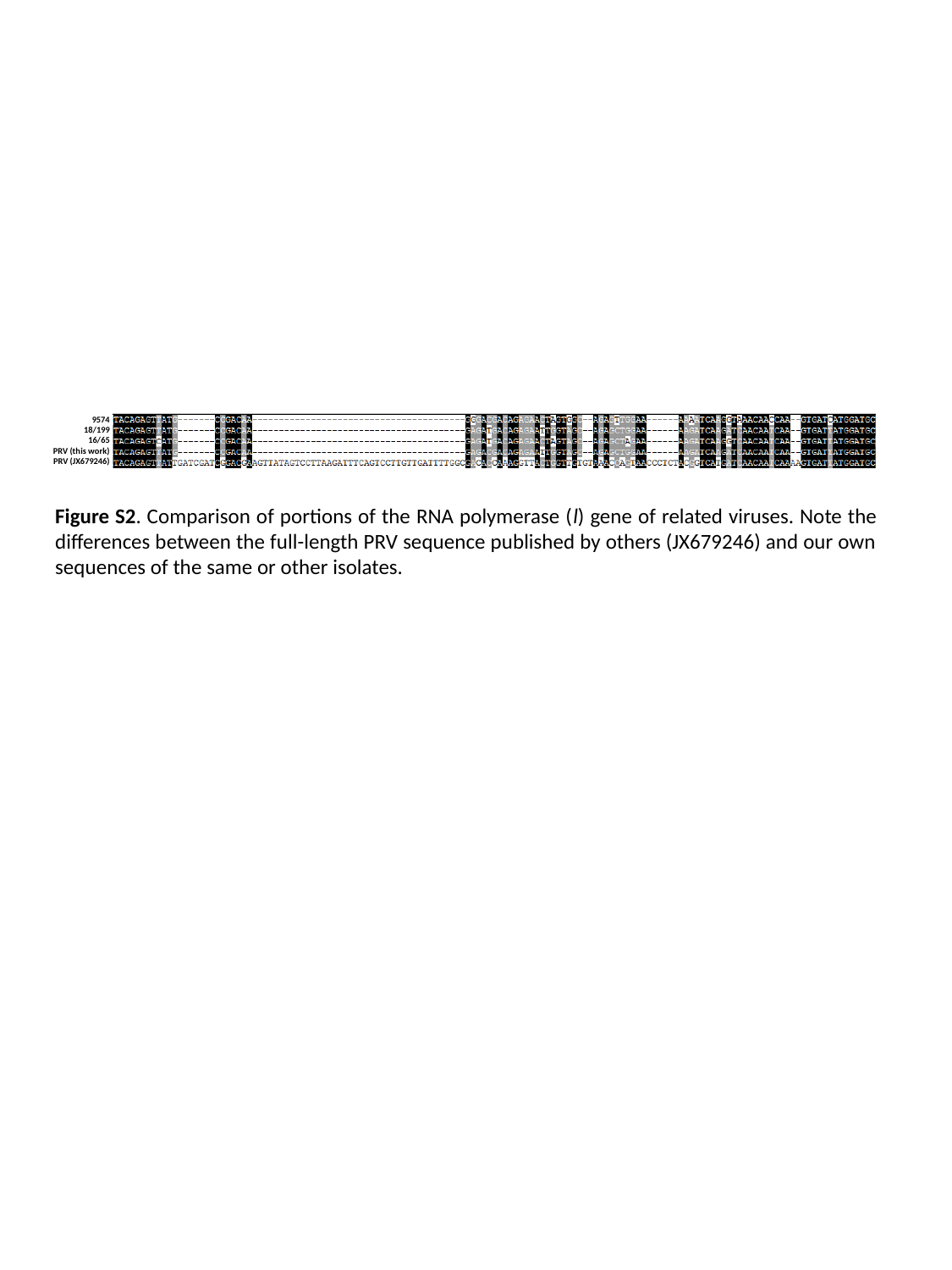

9574
18/199
16/65
PRV (this work)
PRV (JX679246)
Figure S2. Comparison of portions of the RNA polymerase (l) gene of related viruses. Note the differences between the full-length PRV sequence published by others (JX679246) and our own sequences of the same or other isolates.
