# Supplementary material for: Revisiting the Classification of Percid Perhabdoviruses Using New Full-Length Genomes
Source: Viruses. 2020 Jun 16;12(6):649. doi: 10.3390/v12060649 (PMC7354598; doi:10.3390/v12060649)
Supplement: Supplementary file 1 [file viruses-12-00649-s001.zip › pallandre Table S2.pptx]

## Slide 1
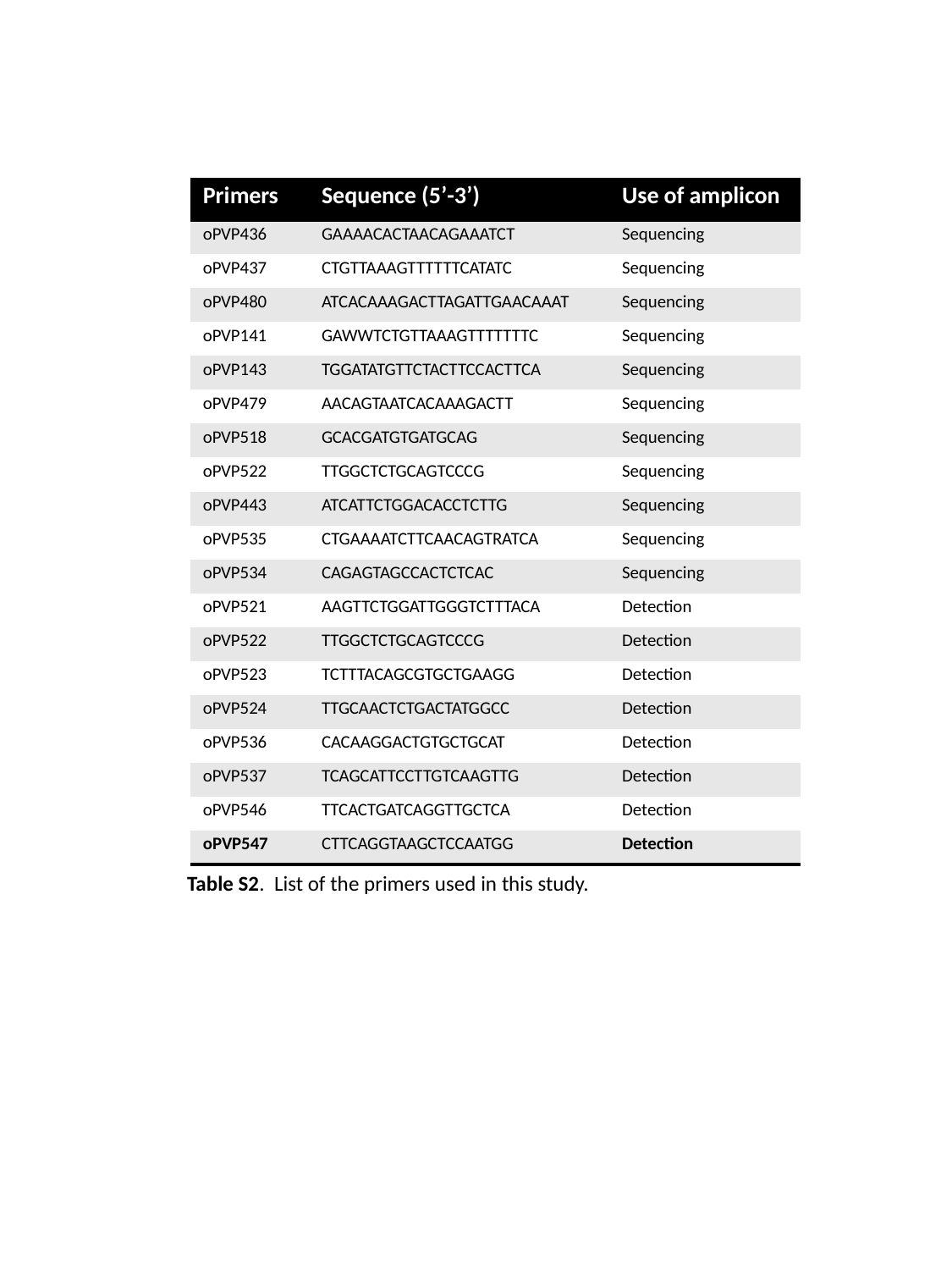

| Primers | Sequence (5’-3’) | Use of amplicon |
| --- | --- | --- |
| oPVP436 | GAAAACACTAACAGAAATCT | Sequencing |
| oPVP437 | CTGTTAAAGTTTTTTCATATC | Sequencing |
| oPVP480 | ATCACAAAGACTTAGATTGAACAAAT | Sequencing |
| oPVP141 | GAWWTCTGTTAAAGTTTTTTTC | Sequencing |
| oPVP143 | TGGATATGTTCTACTTCCACTTCA | Sequencing |
| oPVP479 | AACAGTAATCACAAAGACTT | Sequencing |
| oPVP518 | GCACGATGTGATGCAG | Sequencing |
| oPVP522 | TTGGCTCTGCAGTCCCG | Sequencing |
| oPVP443 | ATCATTCTGGACACCTCTTG | Sequencing |
| oPVP535 | CTGAAAATCTTCAACAGTRATCA | Sequencing |
| oPVP534 | CAGAGTAGCCACTCTCAC | Sequencing |
| oPVP521 | AAGTTCTGGATTGGGTCTTTACA | Detection |
| oPVP522 | TTGGCTCTGCAGTCCCG | Detection |
| oPVP523 | TCTTTACAGCGTGCTGAAGG | Detection |
| oPVP524 | TTGCAACTCTGACTATGGCC | Detection |
| oPVP536 | CACAAGGACTGTGCTGCAT | Detection |
| oPVP537 | TCAGCATTCCTTGTCAAGTTG | Detection |
| oPVP546 | TTCACTGATCAGGTTGCTCA | Detection |
| oPVP547 | CTTCAGGTAAGCTCCAATGG | Detection |
Table S2. List of the primers used in this study.
