# Supplementary material for: Revisiting the Classification of Percid Perhabdoviruses Using New Full-Length Genomes
Source: Viruses. 2020 Jun 16;12(6):649. doi: 10.3390/v12060649 (PMC7354598; doi:10.3390/v12060649)
Supplement: Supplementary file 1 [file viruses-12-00649-s001.zip › pallandre table S3 identities ORF.pptx]

## Slide 1
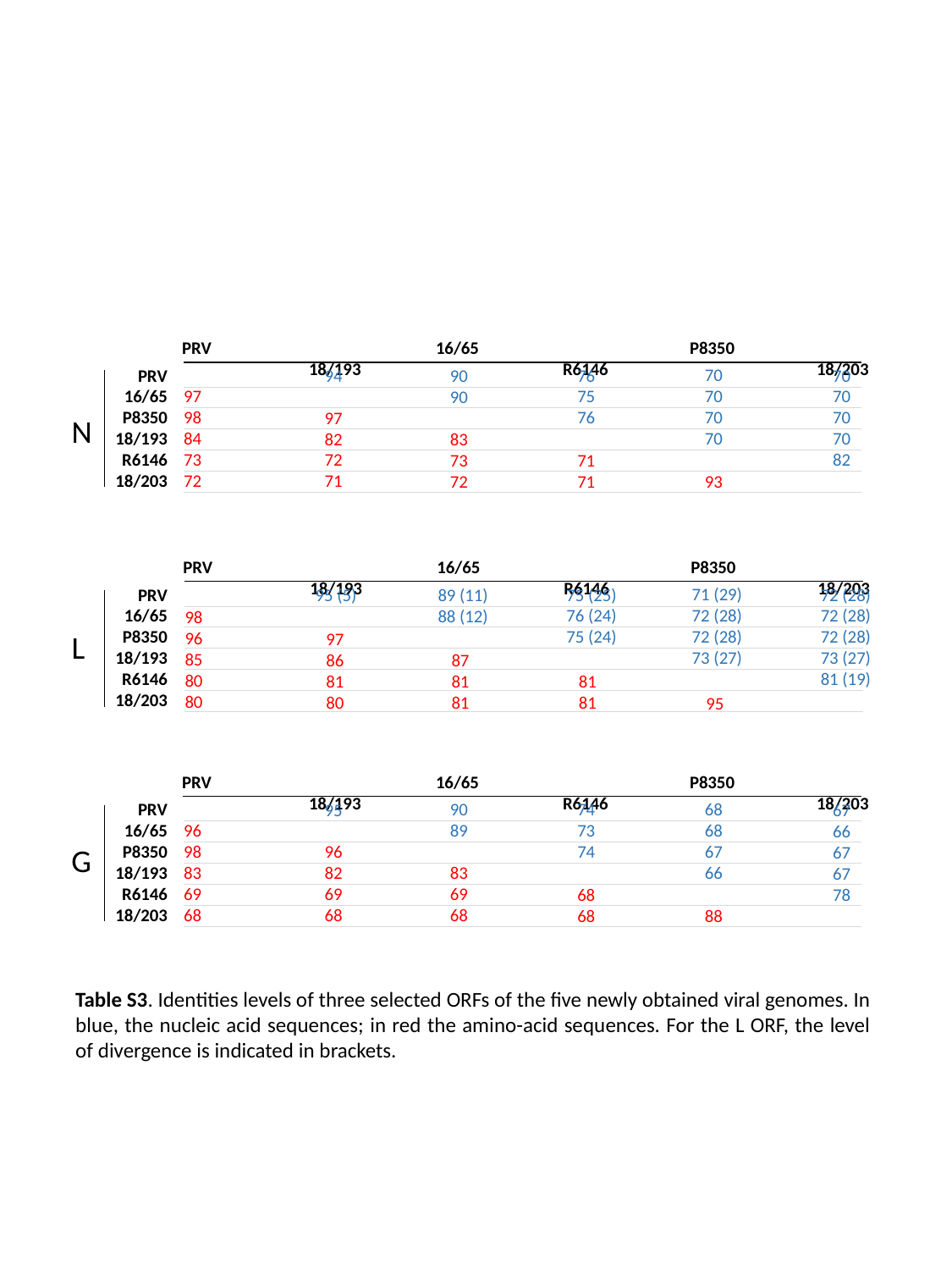

PRV		16/65		P8350		18/193		R6146		18/203
PRV
16/65
P8350
18/193
R6146
18/203
97
98
84
73
72
76
75
76
70
70
70
70
70
70
70
70
82
90
90
94
97
82
72
71
83
73
72
N
71
71
93
PRV		16/65		P8350		18/193		R6146		18/203
PRV
16/65
P8350
18/193
R6146
18/203
75 (25)
76 (24)
75 (24)
71 (29)
72 (28)
72 (28)
73 (27)
72 (28)
72 (28)
72 (28)
73 (27)
81 (19)
89 (11)
88 (12)
95 (5)
98
96
85
80
80
97
86
81
80
L
87
81
81
81
81
95
PRV		16/65		P8350		18/193		R6146		18/203
PRV
16/65
P8350
18/193
R6146
18/203
96
98
83
69
68
74
73
74
68
68
67
66
90
89
67
66
67
67
78
95
96
82
69
68
G
83
69
68
68
68
88
Table S3. Identities levels of three selected ORFs of the five newly obtained viral genomes. In blue, the nucleic acid sequences; in red the amino-acid sequences. For the L ORF, the level of divergence is indicated in brackets.
